# Evolution of dosimetric quantities of International Commission on Radiological Protection (ICRP): Impact of the forthcoming recommendations

**DOI:** 10.4103/0971-6203.35719

**Published:** 2007

**Authors:** A. S. Pradhan

**Affiliations:** C/o. AMPI, RP & AD, CT & CRS, BARC, Mumbai - 400 094, India. E-mail: ambika.s.pradhan@gmail.com

In the previous issue of this journal, Journal of Medical Physics (JMP), there was a news item[1] ‘International Commission on Radiological Protection approves New Fundamental Recommendations on Radiological Protection.’ This appears to have made the readers of JMP curious about the changes in the forthcoming recommendations of the International Commission on Radiological Protection (ICRP). One of the basic concerns of the physicists has always been the quantities and the units of radiation for both measurements and conceptual understanding. This editorial throws some light on the evolution of dosimetric quantities of ICRP and the possible impact of the forthcoming ICRP recommendations on the dosimetric quantities.

A need for pragmatic dosimetric quantities and units for measurements of radiation was realized following the discovery of X-rays in November 1895 and that of radioactivity in February 1896. The available basic quantities were considered inadequate for the purpose. Various proposals, including skin erythema, were mooted to quantify radiation for the realization of the harmful effects of radiation. One of the major milestones in the path of the progress of the International Commission on Radiological Protection (ICRP) had been the adoption of ‘roentgen’ as exposure quantity (unit R) in 1937, as recommended by the International Commission on Radiation Units and Measurements (ICRU). The quantity ‘roentgen’ was based upon the ionization produced by X and gamma rays in the air. To obtain the energy absorbed in a medium, ‘absorbed dose, D’ with unit as ‘rad’ (1 rad = 100 erg) was developed in 1953; and this quantity, absorbed dose in tissue, was adopted[2] as a protection quantity. Although ‘absorbed dose’ still continues to provide the most robust basic quantity, yet it lacked information on the risk due to exposure to radiation because of the variations in radiosensitivity of different tissues/ organs; different types of radiations; and the varying effects of age, sex, time since exposure, etc., for the same amount of absorbed dose. In 1962, a quantity ‘Dose equivalent, DE’ (DE=D.QF.DF…) with unit as ‘rem’ (derived from the earlier defined term ‘roentgen-equivalent man’) was introduced and later adopted.[3] The term ‘quality factor, QF’ was the linear-energy-transfer (LET)-dependent factor by which absorbed doses were to be multiplied to obtain a quantity that expresses on a common scale for all ionizing radiations the irradiation incurred by exposed persons. The dose distribution factor ‘DF’, and the other modifying factors were of little consequence.

The next major milestone came through the 1977 recommendations of ICRP (Pub-26).[4] These recommendations brought in not only the concept of ‘quantified risk’-based approach but also introduced new nomenclature for the protection quantities and the units. The most important part of Pub-26 was the adoption of the weighted whole-body dose equivalent (which led to a new term ‘Effective dose equivalent’) concept for limiting occupational exposures. This approach reflected the increased understanding of the differing radiosensitivity of various organs and tissues and was intended to sum exposures from external sources and from internally deposited nuclides. The symbols of terms ‘dose equivalent,’ ‘quality factor’ and other related factors were replaced by new symbols ‘H,’ ‘Q’ and ‘N’ respectively. (N was later dropped out.) Also, units ‘rad’ and ‘rem’ were virtually replaced by ‘Gray’ (Gy) (1 Gy = 100 rad = 1 J/kg) and ‘Sievert’ (Sv) (1 Sv =100 rem = 1 J/kg). ‘Effective dose equivalent, H_E_’ was the sum of the products of dose equivalents ‘H’ and ‘w_T_’ (a weighting factor representing the ratio of the stochastic risk resulting from a tissue/ organ (T) to the total risk, when the whole body is irradiated uniformly) over the different organs and tissues of the body. The unit of exposure ‘R’ (R = dQ/dm) was discontinued; however, exposure continued to be expressed as charge (coulomb, C) per unit mass of air (1 R = 2.58 × 10^-4^ C/kg) and to a certain extent was also represented by another quantity KERMA (kinetic energy released per unit mass, with unit as Gy) for the indirectly ionizing radiation – namely X and gamma rays and neutrons.[4] Pub-26 provided a good conceptual basis; however, both H and H_E_ were obviously not measurable as the absorbed doses and the quality factors of different types of radiations at all points of all tissues or organs of relevance to protection cannot be measured. A need for measurable quantities was therefore realized where the readouts of the instruments and the dosimeters could be related to both the basic quantities (absorbed dose, fluence or kerma) and the protection quantities (dose equivalent and effective dose equivalent). Consequently, a unified concept of operational quantities for all types of radiations leading to direct additivity of the measured values was developed in 1985 by ICRU.[5] The operational quantities were defined in terms of a receptor (ICRU sphere for area monitoring and the human body for individual monitoring) for both the penetrating and less-penetrating radiations. These, for area monitoring, were ‘Ambient dose equivalent, H* (d)’ and “Directional dose equivalent, H'(d, Ω)”; and for individual monitoring, these were ‘Individual dose equivalent penetrating, Hp(d)’ and ‘Individual dose equivalent superficial, Hs(d)’. ICRU operational quantities were further developed,[6] and the new quantity ‘personal dose equivalent, Hp(d)’ replaced the two former quantities ‘Individual dose equivalent penetrating, Hp(d)’ and ‘Individual dose equivalent superficial, Hs(d)’ for individual monitoring.

By the time, the ICRU operational quantities were to take off, in 1991, ICRP published its new recommendations (Pub-60).[7] A concept of averaging of dose and the mean quality factor over the volume of a specified tissue/ organ due to a type of radiation was introduced. New protection quantities, viz., ‘Equivalent dose’ (H_T_ = Σ_R_ w_R_.D_TR_, where ‘w_R_’ is the radiation weighting factor, which is the same for all organs/ tissues; and D_TR_ is the averaged dose over the tissue/ organ for a radiation type) and ‘Effective dose’ (E= Σ_T_ w_T_.H_T_, where w_T_ is the tissue weighting factor, the same for all types of radiation),' apparently replaced the quantities ‘Dose equivalent, H’ and ‘Effective dose equivalent, H_E_’ of Pub-26. The number of organs/ tissues considered for w_T_ rose to 22 (12+10_Remainders_) in Pub-60 as against 11 (6+5_Remainders_) in Pub-26, with some changes in the values. Also, the values of the radiation weighting factors ‘w_R_’ were considerably different from the values of the quality factors ‘Q’ of Pub-26, especially for neutrons of different energies. Dose equivalent ‘H’ of Pub-26 and Equivalent dose ‘H_T_’ of Pub-60 were different both in the definitions and in the concepts. ‘H’ is a point quantity (product of absorbed dose ‘D’ and the quality factor ‘Q’ at a point in tissue); whereas ‘H_T_’, where the word ‘equivalent’ is an adjective, is related to an averaged effect in an organ/ tissue for different types of radiations. The same was true for the quality factor ‘Q’ and the radiation weighting factor ‘w_R_,’ where the former is related to LET of the radiation and the latter represents the relative biological effectiveness of different radiations. However, Q-L relationship continued to be used for arriving at w_R_. These changes needed a revalidation of the operational quantities and their relationship with the newer protection quantities. A joint group of ICRP and ICRU[8] subsequently brought out the revised conversion coefficients for use in radiological protection against external radiation.

In the forthcoming ICRP recommendations,[9] the definitions and the main concepts of the dosimetric quantities appear to remain unchanged but the development of details of the concepts is expected to make them more meaningful.[10] The emphasis that the quantities ‘Effective dose, E’ and ‘Dose equivalent, H_T_’ (both having the unit ‘Sv’) are relevant only at the lower doses (< 100 mSv) of interest in radiation protection (values of w_R_ used for H_T_ are for RBE_max_ at low doses) will enable the avoidance of misuse of these quantities. The use of ‘Effective dose’ is to be limited to the regulatory compliance of the dose limits for workers and general public through the prospective dose assessment for planning and optimization and/ or through the retrospective dose assessment. For higher doses of relevance to ‘Tissue Reaction’ (the term ‘Deterministic Effect’ to be replaced by ‘Tissue Reaction’?), absorbed dose (Gy) is to be used (not the Dose equivalent) for low LET radiation; and in the case of high LET radiation exposures, an appropriate RBE value is to be used and stated along with the absorbed dose. As reviewed elsewhere,[11] a value of Effective dose does not refer to the actual dose received by any individual; and therefore, it should not be used for any retrospective assessment of individual risk of stochastic effects from radiation exposure, because it is arrived at by using the highly simplified judgments on w_R_ and w_T_ values or estimated on a phantom by using conversion coefficients, dose coefficients and biokinetic and dosimetric models in a reference phantom representing human body of a Reference Person. For the risk assessment, many specific details are needed. Also, Effective dose is not to be used for epidemiological evaluations, medical surveillance (risk-benefit assessment or planning the exposure of patients) or treatment. Similarly, the quantity ‘Collective effective dose’ has been simplified; and all other terms like ‘Collective dose,’ ‘Collective equivalent dose,’ etc., are now dropped out. The purpose of Collective effective dose is limited to optimization and to assessment of technologies and protection options and in no case to be used for predicting the number of cancer deaths due to trivial exposures in a large population or for epidemiological risk assessments and risk projection. In the case of operational quantities, there appears to be a minimal change with regard to ascertaining the value of depth (d) in the ICRU sphere or the human body for area monitoring and individual monitoring respectively and reducing the importance/ need of Personal dose equivalent, Hp(d=3), for the lens of eye. The adopted operational quantities in the new recommendations are Ambient dose equivalent ‘HF*(10)’ and Directional dose equivalent “H'(0.07,Ω)” for area monitoring and Personal dose equivalents ‘Hp(10)’ and ‘Hp(0.07)’ for individual monitoring. H*(10) and Hp(10) are to be used for the assessment of Effective dose (E) and “H'(0.07,Ω)” or ‘Hp(0.07)’ for the assessment of Equivalent doses (H_T_) to the tissue and organs, namely, skin and extremities.

The most striking feature of the forthcoming ICRP recommendations appears to be the updating of the radiation and tissue weighting factors. For X and gamma ray dosimetry, no significant impact is anticipated. For high LET radiation, though the concept of w_R_ is the same as that of Pub-60, the values have now been changed significantly. For neutrons of energy less than 1 MeV, a significant reduction in w_R_ values through a continuous equation has been recommended, mainly due to the consideration of increased contribution to dose by photons produced by lower-energy neutrons in the body/ anthropomorphic phantom (rather than the ICRU sphere considered in the earlier recommendations). The suggested reduction in w_R_ values for protons from 5 to 2 is mainly out of considerations of ranges of protons of pragmatic energies. Also, the sex-averaged values of tissue weighting factors ‘w_T_’ have been recommended for the organs and tissues. These are based on the latest available scientific information on radiobiology of radiation exposure and the adoption of more rational methodology leading to revised risk coefficients. The number of organs/ tissues considered for w_T_ in the forthcoming recommendations would go up to about 28 (14+{13/14}_Remainders_) as against 22 (12+10_Remainders_) in Pub-60. A significant increase from 0.05 to 0.12 in the values of w_T_ for breast, a remarkable decrease from 0.20 to 0.08 for gonads, inclusion of several new organs/ tissues [salivary glands, extrathoracic (ET) region, gallbladder, heart, lymphatic nodes, oral mucosa, prostate, etc.) for the consideration of w_T_; and the concept of equal importance to all the 13 tissues under the new remainder with rationalized sex averaging are bound to cause an impact on the calculation of doses due to exposure to radiation. The fact that there has been no direct evidence that exposure of parents to radiation leads to excess hereditable diseases in offspring probably made ICRP to reevaluate the heritable risks. ICRP still judges that there is some compelling evidence that radiation causes heritable effects in experimental animals. In spite of a remarkable reduction (6- to 8-fold) in the heritable risk coefficients for the heritable risk proposed by ICRP, it continues to emphasize that this reduction in the gonadal tissue weighting factor provides no justification for allowing controllable gonadal exposure to increase in magnitude. Reference Male and Reference Female Voxel phantoms are recommended to be used for dose calculations. The main contributors to the impact are the changes in w_R_ values for neutrons and protons and the values of w_T_ for several organs. A need for the revision of conversion coefficients for external dosimetry and dose coefficients for internal dosimetry is bound to arise. These changes will have far-reaching consequences, and the new recommendations are expected to provide an avenue for further research leading to a large number of publications on the relationship between protection and operational quantities in the coming years.
